# Supplementary material for: The Hippocampal Response to Acute Corticosterone Elevation Is Altered in a Mouse Model for Angelman Syndrome
Source: Int J Mol Sci. 2022 Dec 24;24(1):303. doi: 10.3390/ijms24010303 (PMC9820460; doi:10.3390/ijms24010303)
Supplement: Supplementary file 1 [file ijms-24-00303-s001.zip › ijms-2074275-supplementary_EV.pdf]

Supplementary figures

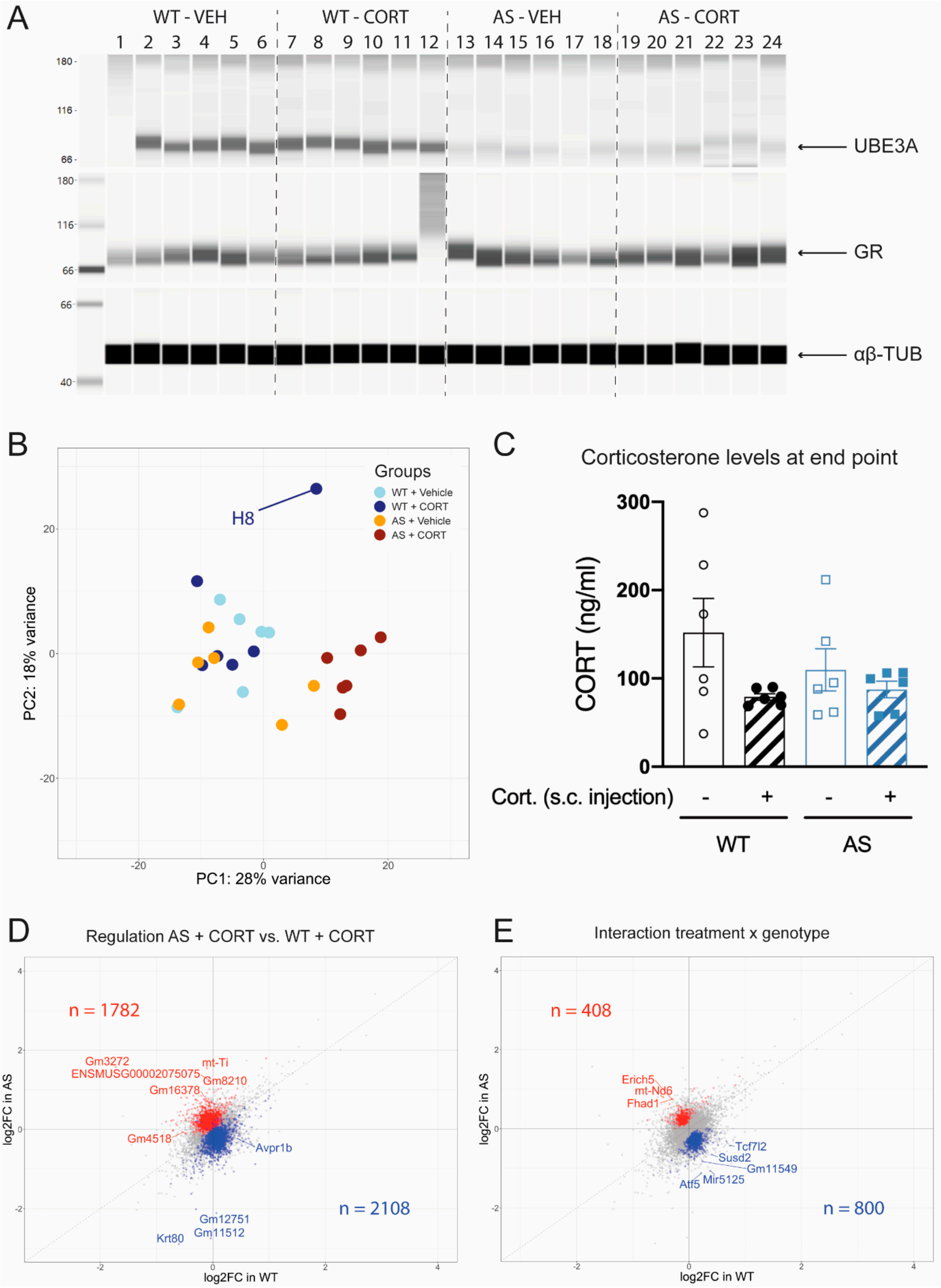

Figure S1. Consequences of acute corticosterone exposure in the AS mouse brain. (A) Protein expression of UBE3A, GR and αβ-tubulin (αβ-TUB) in WT and AS mouse brain after acute treatment

with vehicle or 3mg/kg corticosterone. **(B)** Sample H8 explains 18% of the RNA-seq variance. **(C)** Endpoint corticosterone plasma levels (ng/mL) measured from mouse trunk blood. **(D)** Fold change-fold change plot summarizing the contrast between AS and WT mice treated with acute corticosterone. **(E)** Fold change-fold change plot displaying the genes that significantly contributed to the differential response to acute corticosterone in AS mouse hippocampus.

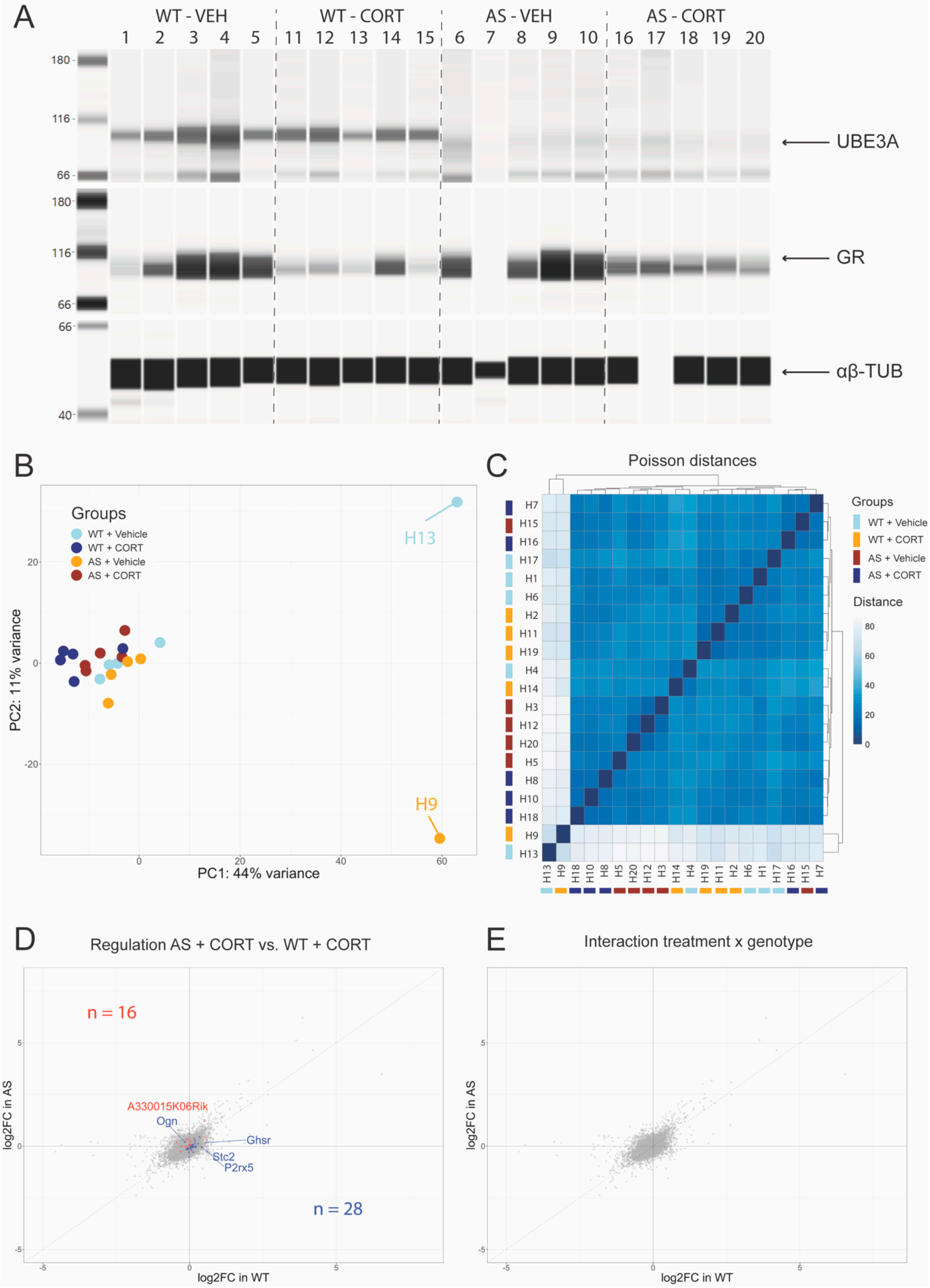

Figure S2. Consequences of continuous corticosterone exposure in the AS mouse brain. (A) Protein expression of UBE3A, GR and αβ-tubulin (αβ-TUB) in WT and AS mouse brain after continuous treatment with vehicle or corticosterone (20 mg releasing-pellet). **(B)** Samples H9 and H13 biased the variance analysis. **(C)** Samples H9 and H13 were technical outliers as determined by the Poisson distance analysis. **(D)** Fold change-fold change plot summarizing the contrast between AS and WT mice treated with continuous corticosterone. **(E)** Fold change-fold change plot displaying the genes that significantly contributed to the differential response to continuous corticosterone in AS mouse hippocampus.

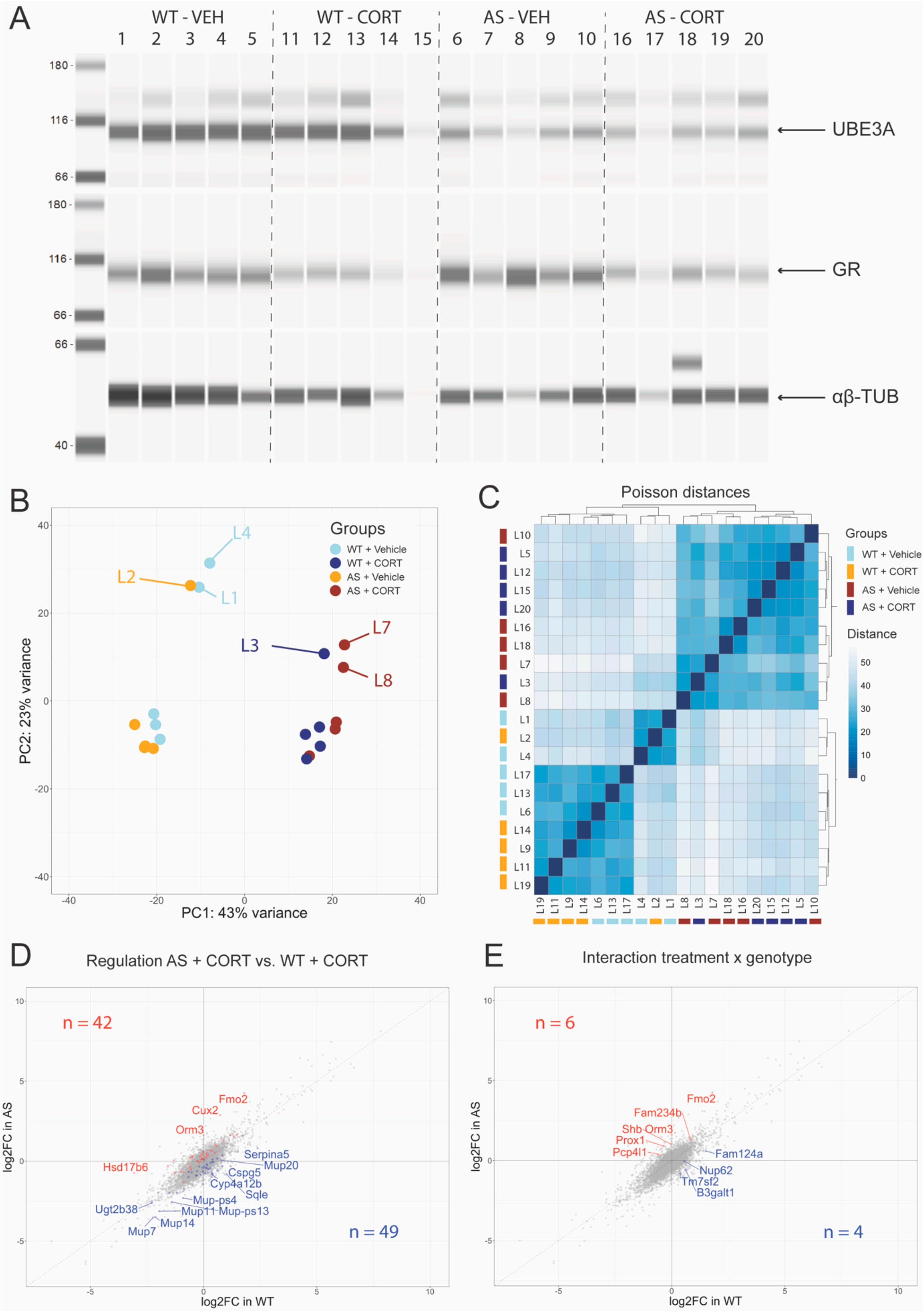

Figure S3. Consequences of continuous corticosterone exposure in the AS mouse liver. (A) Protein expression of UBE3A, GR and αβ-tubulin (αβ-TUB) in WT and AS mouse liver after continuous treatment with vehicle or corticosterone (20 mg releasing-pellet). **(B)** Biological variability between

mice contributed to 23% of the RNA-seq variance. **(C)** The Poisson distance analysis did not highlight specific outliers. **(D)** Fold change-fold change plot summarizing the contrast between AS and WT mice treated with continuous corticosterone. **(E)** Fold change-fold change plot displaying the genes that significantly contributed to the differential response to continuous corticosterone in AS mouse liver.

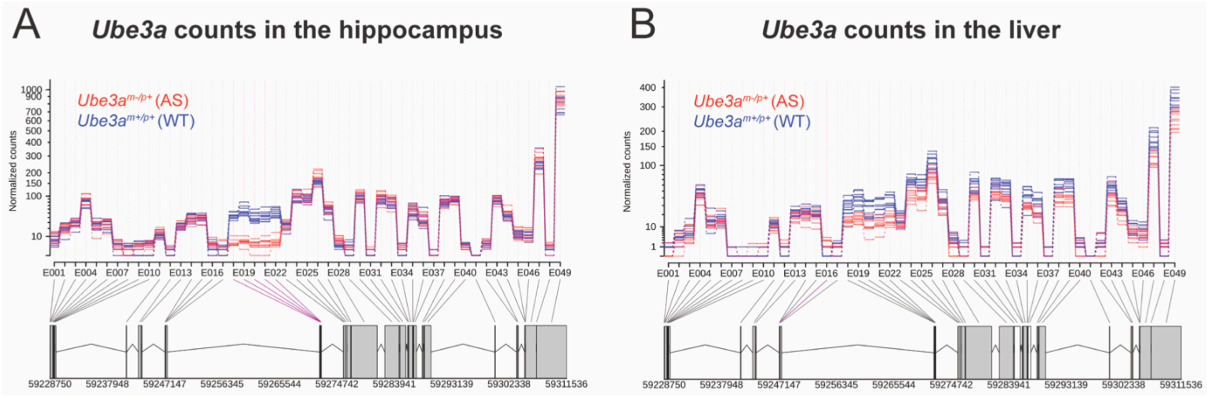

Figure S4. Differential exon usage analysis of *Ube3a*. **(A)** Exon usage plot of *Ube3a* in the AS mouse hippocampus, **(B)** and liver.

Supplementary tables

Table S1. MARCoNI output.

Table S2. Output of RNA-seq differential gene expression analyses in the mouse hippocampus and liver tissues.

Table S3. Results of gene ontology and pathway enrichment analyses.
